# Phylogeographic analysis of Tula hantavirus highlights a single introduction to central Europe

**DOI:** 10.1093/ve/veac112

**Published:** 2022-12-22

**Authors:** Valentina Cirkovic, Simon Dellicour, Gorana Stamenkovic, Marina Siljic, Ana Gligic, Maja Stanojevic

**Affiliations:** Faculty of Medicine, University of Belgrade, Dr Subotica 8, Belgrade 11000, Serbia; Spatial Epidemiology Lab (SpELL), Université Libre de Bruxelles, CP160/13, 50, av. FD Roosevelt, Bruxelles 1050, Belgium; Department of Microbiology, Immunology and Transplantation, Rega Institute, KU Leuven, Herestraat 49, Leuven 3000, Belgium; University of Belgrade, Institute for Biological Research ‘Siniša Stanković’, Bulevar despota Stefana 142, Belgrade 11108, Serbia; Faculty of Medicine, University of Belgrade, Dr Subotica 8, Belgrade 11000, Serbia; Institute of Virology, Vaccines and Sera Torlak, Vojvode Stepe 458, Belgrade 11000, Serbia; Faculty of Medicine, University of Belgrade, Dr Subotica 8, Belgrade 11000, Serbia

**Keywords:** Hantaviridae, Tula hantavirus, phylogeography, Eurasia

## Abstract

Orthohantaviruses are zoonotic pathogens of humans, unique among the bunyaviruses in not being transmitted by an arthropod vector. Tula orthohantavirus (TULV) is an old-world hantavirus, of yet unclear human pathogenicity, with few reported cases of clinically relevant human infection. So far, phylogeographic studies exploring the global pathways of hantaviral migration are scarce and generally do not focus on a specific hantavirus species. The aim of the present study was to reconstruct the dispersal history of TULV lineages across Eurasia based on S segment sequences sampled from different geographic areas. Maximum-likelihood and Bayesian inference methods were used to perform the phylogenetic analysis and phylogeographic reconstructions. Sampling time and trapping localities were obtained for a total of 735 TULV S segment sequences available in public databases at the time of the study. The estimated substitution rate of the analyzed partial S segment alignment was 2.26 × 10^–3^ substitutions/site/year (95 per cent highest posterior density interval: 1.79 × 10^−3^ to 2.75 × 10^–3^). Continuous phylogeography of TULV S segment sequences placed the potential root and origin of TULV spread in the Black Sea region. In our study, we detect a single-lineage introduction of TULV to Europe, followed by local viral circulation further on.

## 1. Introduction

Orthohantaviruses are among the most important human zoonotic pathogens, associated with a number of different animal host species including rodents, shrews, moles, and bats (de Oliveira et al. 2014; Chen et al. 2019). The members of the family *Hantaviridae*, order *Bunyavirales*, are enveloped three-segmented negative-sense RNA viruses. Hantavirus genomic segments are large—L, medium—M, and small—S, coding for viral polymerase, viral glycoprotein precursor of two envelope glycoproteins (Gn and Gc), and viral nucleocapsid protein, respectively (Plyusnin et al. 1996a). Unlike most bunyaviruses that are arthropod-borne, hantaviruses are transmitted to humans in direct contact with persistently infected reservoir species.

Pathogenic hantaviruses are known to be the etiological agents of two clinical syndromes: hemorrhagic fever with renal syndrome (HFRS) in Eurasia and hantavirus pulmonary syndrome (HPS) in the Americas. Mortality rates of these zoonotic diseases vary, reaching up to 18 per cent for HFRS and 60 per cent for HPS (Gligic 2008; Jonsson et al. 2010; Papa 2012). Over forty hantavirus species are circulating in different natural reservoirs worldwide, of which Dobrava-Belgrade, Seoul, Puumala (PUUV), Hantaan, and Saaremaa are found in Europe, causing mild-to-severe forms of HFRS.

Tula virus (TULV) is an old-world hantavirus considered to be non-pathogenic (Gligic et al. 1988; Vapalahti et al. 2003; Papa 2012). Up to now, the knowledge regarding the human pathogenicity of TULV has been rather sparse, with few reports of human infection (Vapalahti et al. 1996; Schultze et al. 2002; Clement et al. 2003). However, cases of clinically relevant human infection by TULV have recently been reported, highlighting its role as an emerging human pathogen (Zelena et al. 2013; Reynes et al. 2015; Hofmann et al. 2021). Indeed, considerable serologic cross-reactivity between PUUV and TULV could be the underlying reason for the underestimated TULV prevalence and pathogenicity (Clement and Van Ranst 2016).

The evolution and diversification of hantaviruses are considered to have been largely influenced by the migration patterns of both viruses and their hosts. The geographic distribution and ecology of hantaviruses are directly related to the distribution of their natural hosts (Jonsson et al. 2010). Hence, hantaviruses are commonly divided into two groups: old-world hantaviruses, which circulate in Europe and Asia and are carried by species of five different rodent genera (*Apodemus*, *Microtus*, *Myodes*, *Rattus*, and *Arvicola*) and two insectivore families (*Soricidae* and *Talpidae*), and new-world hantaviruses, which circulate in the Americas and are mostly associated with the members of the rodent subfamilies *Neotominae* and *Sigmodontinae*.

The first reports of TULV isolation date from the mid-nineties, from *Microtus arvalis* and *Microtus rossiaemeridionalis* captured in Tula region in Russia (Plyusnin et al. 1994) and from *M. arvalis* (Sibold et al. 1995) captured in west Slovakia, followed by TULV detection in various European countries further on (Plyusnin et al. 1995; Bowen et al. 1997; Sibold et al. 1999; Heyman 2002). A retrospective analysis of the archived samples revealed TULV presence in much earlier rodent samples, including a Serbian isolate from *Microtus subterraneus*, captured in 1987, which remains among the oldest TULV sequences at present, along with the initial ones from Russia (Song et al. 2002).

So far, studies of hantaviral geographic distribution have found an association of different hantaviruses with landscape and geographic features of their host habitat. This association has clearly been observed at the local level in both new- and old-world hantaviruses (Torres-Perez et al. 2011; Korva et al. 2013; Laenen et al. 2016). However, studies using phylogeographic approach to explore the global pathways of hantaviral migration are rather few and do not focus on a particular hantavirus—the existing ones are performed at the level of the whole viral family or genus (Bennett et al. 2014; Souza et al. 2014), with the exception of some insight regarding insectivore-borne hantaviruses and, more recently, PUUV (Ling et al. 2018; Castel et al. 2019; Laenen et al. 2019). In particular, no study has so far explored the spatial spread of TULV.

Despite the increasing number of viral sequences deposited in the National Center for Biotechnology Information (NCBI) database, TULV remains less represented, with just over a thousand available sequences in total, of any length. Moreover, it has been shown that the most robust results are obtained if the input dataset is made of full-length genomic sequences (Dudas and Bedford 2019). However, in view of the segmented nature of their genome, the number of complete TULV genome sequence sets, especially of all the three genetic segments from the same source, is still even scarcer, whereas the S genomic segment has been sequenced the most often.

In the present study, we took advantage of a comprehensive dataset made of S segment sequences isolated from different geographic areas to apply a Bayesian phylogeographic framework with the aim to reconstruct the dispersal history of TULV lineages across the Palearctic region.

## 2. Methods

### 2.1 Study dataset

A detailed NCBI database search performed in April 2022 revealed 764 TULV S segment sequences of different length and genomic position (http://www.ncbi.nlm.nih.gov/nuccore), which were included in the initial alignment by ClustalW algorithm implemented in MEGA X software package (Kumar et al. 2018). Upon manual editing and inspection of the resulting alignment, 735 sequences were selected for the study, based on the optimal alignment length with maximal number of available sequences. The resulting alignment was of 543 nucleotides (nt) in length, corresponding to nt positions 418–960 of TULV S segment reference sequence. Of note, the dataset encompassed a range of the S segment that has been most commonly used for TULV studies so far (Saxenhofer et al. 2017; Jeske et al. 2021; Schmidt et al. 2021). One sequence was excluded upon screening for recombination by using methods implemented in the RDP4 program (Martin and Rybicki 2000); hence, the alignment for the initial phylogenetic tree reconstruction contained 734 TULV S segment sequences. Further on, based on zero genetic distance, 322 identical sequences were removed, resulting in the final dataset of 412 TULV S segment sequences. The dataset comprised sequences of both animal and human origin sources, collected between 1987 and 2018 in 188 different locations across 17 countries within Europe and central Asia. Latitude and longitude coordinates for each sample were retrieved from the sampling locations, as reported in the NCBI database or directly from the related publications.

### 2.2. Phylogenetic analysis

jModeltest 0.1.1 software was used to select the best-fitting substitution model using all eighty-eight proposed models (Posada 2008). Based on the best Akaike information criterion score, the best-fitting model for the present dataset was general time reversible with gamma-distributed rates among sites and proportion of invariant sites (GTR + G + I). Maximum-likelihood (ML) and Bayesian statistical approaches were employed to infer the evolutionary relationship of analyzed sequences, using MEGA X and BEAST 1.10.4, respectively (Kumar et al. 2018; Suchard et al. 2018).

We used the likelihood-mapping method implemented in the program TreePuzzle (Strimmer and von Haeseler 1997) to first explore the phylogenetic signal associated with the sequence alignment.

Temporal signal was examined by the root-to-tip regression approach implemented in the program TempEst (Rambaut et al. 2016). ML phylogenetic tree previously generated in MEGA X using the GTR + I + G substitution model was used as an input for this analysis. To further assess the extent of temporal structure, we employed a Bayesian date-randomization test (DRT), in which sampling dates are randomly reassigned to the sequences and the analysis of the data is repeated a number of times in order to generate a set of rate estimates from date-randomized data (Ramsden et al. 2009).

The amount of phylogenetic information contained in a sequence alignment may be influenced by base substitution saturation to the level of disrupting analyses involving deep phylogeny. The level of substitution saturation in the dataset was assessed using the substitution saturation test implemented in the software package DAMBE6 (Xia 2018). The results were presented as scatter plots of pairwise nt transition (s) and transversion (v) substitutions against the Tamura and Nei 1993 genetic distance.

### 2.3 Molecular clock and phylogeographic analyses

The estimation of substitution rate and phylogeographic history was performed using the software package BEAST 1.10.4 (Suchard et al. 2018). A molecular clock-based phylogenetic analysis was performed, using the best-fitting nt substitution model identified earlier, an uncorrelated lognormal relaxed molecular clock model, and a Bayesian skygrid tree prior. The Markov Chain Monte Carlo (MCMC) algorithm was run for more than 2.5 × 10^8^ generations, sampling every 70,000 generations. Moreover, we used a codon partition (the ‘codon positions 1, 2, and 3’ option), so that each codon position had its own evolutionary rate. Statistical support for specific clades was obtained by calculating the posterior probability of each monophyletic clade. MCMC convergence was assessed with Tracer v 1.7 on the basis of the effective sampling size (>200) estimated for each parameter (Rambaut et al. 2018). Finally, the maximum clade credibility (MCC) tree was generated with TreeAnnotator 1.10.4 (Suchard et al. 2018).

The BaTS program (http://evolve.zoo.ox.ac.uk/Evolve/BaTS.html) was used to investigate the strength of the spatial association with the dispersal history of TULV lineages. This program provides an appropriate method to calculate the degree of correlation, using different statistics including the association index (AI) and parsimony score (PS) (Parker, Rambaut, and Pybus 2008). Both statistics were calculated based on posterior sets of trees, obtained through earlier Bayesian MCMC analysis. The parameters for this analysis were the number of states (in our case 188) and the number of replicates (100). If the obtained *P*-value was less than 0.05, the null hypothesis was rejected.

Continuous phylogeographic inference was performed using the relaxed random walk (RRW) diffusion model implemented in BEAST 1.10.4 and with the BEAGLE library (Ayres et al. 2012) to improve computational performance. The Cauchy RRW model was the best-chosen model to reconstruct the dispersal history along time-scaled genealogies. Spatiotemporal information embedded in inferred phylogenetic trees was subsequently extracted with the R package ‘seraphim’ (Dellicour, Rose and Pybus 2016a; Dellicour 2016b). We then used the ‘spreadGraphic2’ function of that package for visualizing the outcome of the continuous phylogeographic reconstruction and the ‘spreadStatistics’ function to estimate the mean and weighted lineage dispersal velocity.

## 3. Results and discussion

**Figure 1.**
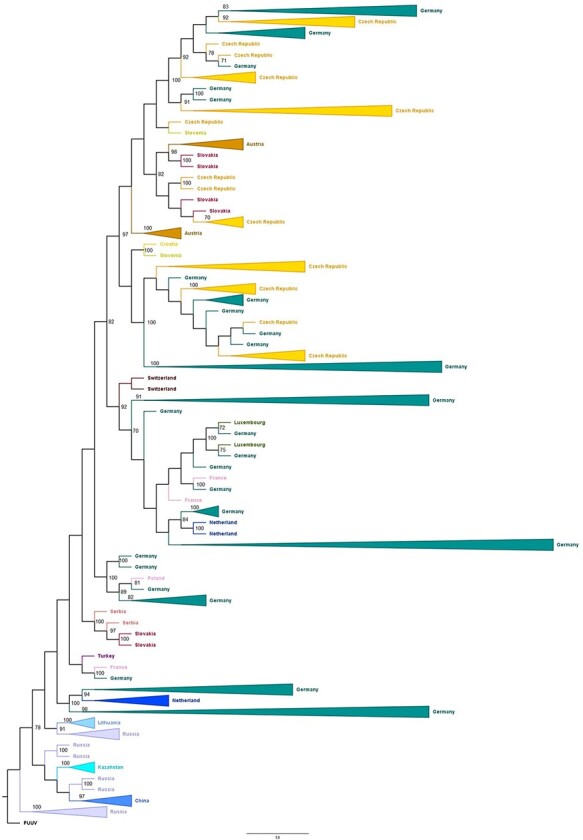
ML phylogenetic tree generated by MEGA X software based on 543 nt S segment of 734 examined TULV sequences. In order to get better visibility of the tree, each branch was colored in accordance with the respective country. Clades consisting of three or more sequences originating from the same country are compressed. The numbers in bifurcations indicate bootstrap values (a value of >65 per cent was considered as strong nodal support). The tree was rooted with the PUUV reference strain NC005224.

ML phylogenetic analysis based on the dataset comprising 734 TULV S segment sequences inferred mostly yet not exclusively geographically related clustering (Fig. 1). Samples from Russia are the most basal in the tree, whereas sequences from central (Austria, the Czech Republic, Slovakia, Croatia, and Slovenia) and Western Europe (Germany, Switzerland, Luxembourg, France, and the Netherlands) tend to cluster together. The tree was rooted with PUUV reference strain NC005224.

A distinct clade was formed by four strains previously already described as recombinants: two recombinant sequences were from eastern Slovakia (Sibold et al. 1999), where the host animals (*M. arvalis*) had been trapped in 1995 in Kosice region, and two from Serbia: one from *M. subterraneus* captured in 1987 in west Serbia and the other from *M. arvalis* captured in 2007 in central Serbia (Song et al. 2002; Nikolic et al. 2014). In view of the same pattern of recombination and considerable time span between animal trapping of 20 years, we speculated that this TULV variant has become a stable circulating compartment of virus population, justifying their inclusion into the analysis.

The assessment of phylogenetic noise of the studied dataset of TULV S segment sequences, through investigation of 10,000 randomly chosen quartets by means of likelihood mapping, showed that only 0.3 per cent fell in the central area of the likelihood map and 99.7 per cent were at the corners of the triangle, implying sufficient phylogenetic information (Supplementary Fig. S1A). The assessment of the temporal signal associated with the sequence alignment through root-to-tip regression analysis revealed a correlation coefficient of 0.24. However, DRT results implied that the temporal signal in the analyzed TULV S segment data is sufficient to reliably estimate the substitution rate of the given data, considering the fact that the 95 per cent highest posterior density (HPD) of the substitution rate did not overlap between the true and randomized data. The saturation analysis plot indicated no saturation for closely related sequences compared to slight saturation for more distantly related sequences (Supplementary Fig. S1B). This result implied that the molecular clock analysis may underestimate the real age of the deepest splits of the phylogeny. In view of this finding, the obtained estimated time to the most recent common ancestor of >190 years ago (1,826.9, 95 per cent HPD = [1,752.2–1,887.5]) may reflect saturation among the distantly related sequences. The obtained estimate of a substitution rate of 2.26 × 10^–3^ substitution/site/year (95 per cent HPD interval: 1.79 × 10^−3^ to 2.75 × 10^–3^) is in line with the previous studies, which ranged from 1.99 × 10^–2^ to 8.87 × 10^–3^ substitutions/site/year (Ramsden et al. 2008). Hantaviruses have long been considered to had cospeciated with their rodent and insectivore hosts, ever since these mammals shared their last common ancestor approximately 100 million years ago (Ramsden et al. 2009). This notion was based on the phylogenetic inference of the *Hantavirus* genus members that revealed three constantly well-defined clades, each associated with one of the three subfamilies of Muroid rodents: *Arvicolinae*, *Murinae*, and *Sigmodontinae* (Plyusnin 1996b). However, after the inclusion of hantavirus sequences from insectivores into analyses, the overall phylogenetic inference of both rodent- and insectivore-borne hantaviruses seemingly showed that neither of these formed monophyletic clade (Arai 2008). Following the assumption of codivergence, the rate of molecular evolutionary change in hantaviruses had initially been estimated at approximately 10^–7^ nt substitutions/site/year (Hughes and Friedman 2000; Sironen et al. 2001), which is several orders of magnitude lower compared to the substitution rates obtained for other RNA viruses (10^–2^ to 10^–4^ substitutions/site/year; Jenkins 2002; Hanada, Suzuki, and Gojobori 2004). Substitution rate in hantaviruses is the consequence of many factors regarding the viral life cycle, such as mutation rate, generation time, transmission, and natural selection (Duffy, Shackelton, and Holmes 2008). Hantaviruses possess RNA-dependent RNA polymerase for replication, which lacks the proofreading and repair mechanisms and operates with an error rate of ∼1 mutation/replication/genome (Drake 1999). The time span of 31 years covered in our study is also an important factor for estimation of the long-term evolutionary rates of viruses since sampling over longer time period allows for more accurate substitution rate estimation.

**Figure 2.**
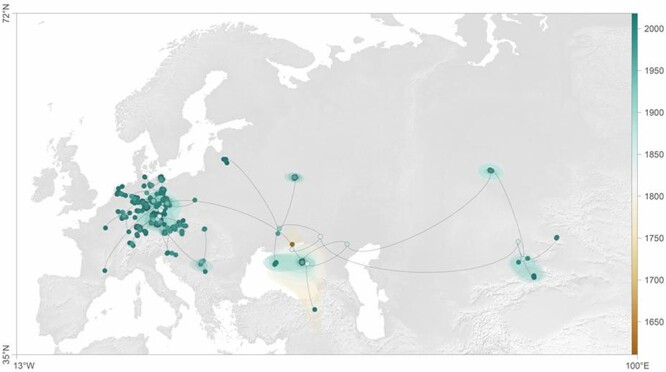
Reconstructed spatiotemporal diffusion history of TULV in Europe and Asia; mapped MCC tree and 80 per cent HPD regions based on 1,000 trees regularly sampled from the post-burn-in posterior distribution of continuous phylogeographic analysis. Nodes of the MCC tree are colored according to their time of occurrence.

Herein, a spatially explicit phylogeographic approach was used to reconstruct the dispersal history and dynamics of TULV lineages. We used the RRW model implemented in BEAST 1.10.4 (Lemey et al. 2010) to gain a realistic picture of the dispersal routes of TULV lineages (Fig. 2), a method of choice that has previously been applied to other zoonotic RNA viruses such as rabies virus and West Nile virus (Pybus et al. 2012; Kuzmina et al. 2013; Zehender 2017). The strength of the spatial association with the TULV transmission pattern throughout Europe and Asia has also been estimated using AI and PC statistics. The analysis showed a very strong geographic clustering of phylogenetically related samples (*P* = 0.000 for both AI and PS statistics) and a high degree of spatial signal. Our analysis estimated the geographic origin of the analyzed viral lineages in the region of north–east Black Sea coast, with a single introductory pathway toward central Europe; upon entry, viral dispersal led to the local circulation of viral lineages, toward the south, to the Balkans, northwards in the direction of the North Sea, and toward the west. In parallel with a within-Europe spread, a dispersal pathway from the initial origin led to east, toward central Asia and Russia. Notably, our phylogeographic analysis highlighted a single-lineage introduction event of TULV to central Europe, with the ensuing complex pattern of local viral circulation within Europe (Fig. 2, Supplementary Fig. S2).

In general, data regarding the origin and dispersal history of different hantaviruses are limited. Previous studies of the general hantaviral phylogeography mostly placed the putative origin of potential hantavirus spread in eastern Asia, with further local and global viral spread, e.g. independent spread to Americas separately in shrews and rodents (Bennett et al. 2014). It has been hypothesized that, initially, these hantaviruses had probably been adapted to the *Myodes* genus and gave rise to viral spread toward northern Europe and further evolution of viruses such as PUUV (Souza et al. 2014).

Factors, such as host behavior and environmental and climate determinants, may affect the geographic distribution, abundance, and dynamics of the carrier rodent species and therefore change the ecology of hantaviruses. Notably, TULV phylogeographic range, as obtained in our study, coincides with the distribution range of *M. arvalis*, considered the main TULV host (https://mol.org/species/Microtus_arvalis). However, TULV is known to be the most ‘promiscuous’ of hantaviruses, as numerous small mammal species have been implicated as its putative reservoirs, including *M. arvalis*, *M. subterraneus*, *M. rossiaemeridionalis*, *M. agrestis, M. gregalis, A. amphibius*, and *L. lagurus* (Schmidt-Chanasit et al. 2010; Schlegel et al. 2012). Broadly, geographic range and phylogeographic structure of TULV reflect those of evolutionary trajectory of its main natural reservoir, common vole (*M. arvalis*), for which speciation processes are commonly linked to the last glacial maximum (Tougard et al. 2008; Braaker and Heckel 2009).

Common voles are subterrestrial mammals that can be found in a variety of habitats including forests, meadows, pastures, and farming areas from sea level up to around 2,000 m altitude in the Alps (Heckel 2005; Braaker and Heckel 2009; Yigit et al. 2016). Voles tend to build subterraneus burrows in their natural habitats, which serve as shelters for colonies of voles during the winter time (Heckel 2005). This type of complex social behavior may increase the possibility of direct TULV transmission among these small rodents, particularly in short distances (Deter 2008). Previous studies implied complex connection between hantavirus natural reservoirs and environmental and climate changes (Dearing and Dizney 2010). Small mammals are very sensitive to climate and habitat changes. Climate changes influence food availability and alter winter conditions, which in turn may affect the distribution of small mammals. The seasonal changes of home range sizes were also observed, resulting in larger home ranges in summer (Wang 2012). Based on a modeling and observational approach, home range size of common voles has been estimated to around 195 m^2^ in males and 140 m^2^ in females (Jacob 2000). Seeing that this species is characterized by limited dispersal ability and small local effective population sizes, the strong genetic structuring among populations at regional and local scales was also observed (Heckel 2005; Schweizer et al. 2007). Of note, opposite to the very restricted home range of common vole and contrary to the theory of geographical barriers separating different evolutionary lineages, the presence of genetically identical mitochondrial DNA on the northern and southern slopes of the Alps has been described (Braaker and Heckel 2009). Yet, a restricted dispersal capacity of voles influences the reduction in the opportunity of TULV dissemination on long distance. Here, we estimated a relatively low mean lineage velocity with a median value of 27.1 km/year (95 per cent HPD = [20.9–46.7]) and a weighted lineage dispersal velocity of 9.1 km/year (95 per cent HPD = [7.4–11.3]). Furthermore, a recent comparative phylogenetic study at a fine geographical scale suggested the existence of a strong barrier for the effective TULV transmission between local host populations in a geographical region in which two distinct evolutionary lineages in the common vole interact and interbreed (a hybrid zone) (Saxenhofer et al. 2019).

## 4. Conclusion

In conclusion, we present the first phylogeographic analysis of TULV, performed on a dataset of 412 TULV S segment sequences sampled from 188 locations. We focus on the evolutionary and dispersal history of TULV lineages in Eurasia using Bayesian inference methods. Phylogeographic analysis included all publicly available unique TULV S segment sequences of the specified region present in the NCBI database at the time of the analysis. Dispersal routes of the studied viral lineages appear to be strongly shaped by geographic distances and highlight a single migration event, leading to lineage introduction to central Europe.

## Supplementary Material

veac112_Supp

## Data Availability

*Sequence data are available in Supplementary Table S1*, which has a list of accession numbers together with the year of collection of sequences used in the study.

## References

[R1] Arai S. (2008) ‘Phylogenetically Distinct Hantaviruses in the Masked Shrew (*Sorex cinereus*) and Dusky Shrew (*Sorex monitcolus*) in the United States’, *American Journal of Tropical Medicine and Hygiene*, 78: 348–51. 18256444 PMC2262799

[R2] Ayres D. L. et al. (2012) ‘BEAGLE: An Application Programming Interface and High-Performance Computing Library for Statistical Phylogenetics’, *Systematic Biology*, 61: 170–3. 21963610 10.1093/sysbio/syr100 PMC3243739

[R3] Bennett S. N. et al. (2014) ‘Reconstructing the Evolutionary Origins and Phylogeography of Hantaviruses’, *Trends in Microbiology*, 22: 473–82. 24852723 10.1016/j.tim.2014.04.008 PMC4135427

[R5] Bowen M. D. et al. (1997) ‘Puumala Virus and Two Genetic Variants of Tula Virus Are Present in Austrian Rodents’, *Journal of Medical Virology*, 53: 174–81. 9334930 10.1002/(sici)1096-9071(199710)53:2<174::aid-jmv11>3.0.co;2-j

[R6] Braaker S. , and Heckel G. (2009) ‘Transalpine Colonisation and Partial Phylogeographic Erosion by Dispersal in the Common Vole (*Microtus arvalis*)’, *Molecular Ecology*, 18: 2518–31. 19389166 10.1111/j.1365-294X.2009.04189.x

[R7] Castel G. et al. (2019) ‘Phylogeography of *Puumala orthohantavirus* in Europe’, *Viruses*, 11: 679. 10.3390/v11080679 PMC6723369 31344894

[R8] Chen J. T. et al. (2019) ‘Identification and Characterization of a Novel Subtype of Tula Virus in *Microtus arvalis* Obscurus Voles Sampled from Xinjiang, China’, *Infection Genetics Evolution*, 75: 104012. 10.1016/j.meegid.2019.104012 31446137

[R9] Clement J. et al. (2003) ‘Human Tula Virus Infection or Rat-Bite Fever?’, *European Journal of Clinical Microbiology and Infectious Disease*, 22: 332–3. 10.1007/s10096-003-0921-7 12736795

[R10] Clement J. and Van Ranst M. (2016) ‘Three Vole Species and One (?) Novel Arvicolid Hantavirus Pathogen: Tula Virus Revisited’, *Euro Surveillance*, 21: pii=30108. 10.2807/1560-7917.ES.2016.21.2.30108 26794642

[R11] de Oliveira C. et al. (2014) ‘Rio Mamore Virus and Hantavirus Pulmonary Syndrome, Brazil’, *Emerging Infectious Diseases*, 20: 1568–70. 25152089 10.3201/eid2009.131472 PMC4178416

[R12] Dearing M. D. , and Dizney L. (2010) ‘Ecology of Hantavirus in a Changing World’, *Annals of the New York Academy of Sciences*, 1195: 99–112. 20536819 10.1111/j.1749-6632.2010.05452.x

[R13] Dellicour S. , Rose R., and Pybus O. G. (2016a) ‘Explaining the Geographic Spread of Emerging Epidemics: A Framework for Comparing Viral Phylogenies and Environmental Landscape Data’, *BMC Bioinformatics*, 17: 82. 10.1186/s12859-016-0924-x PMC4750353 26864798

[R14] Dellicour S. (2016b) ‘SERAPHIM: Studying Environmental Rasters and Phylogenetically-Informed Movements’, *Bioinformatics*, 32: 3204–6. 27334476 10.1093/bioinformatics/btw384

[R15] Deter J. (2008) ‘Kinship, Dispersal and Hantavirus Transmission in Bank and Common Voles’, *Archives of Virology*, 153: 435–44. 18071626 10.1007/s00705-007-0005-6

[R16] Drake J. W. (1999) ‘The Distribution of Rates of Spontaneous Mutation over Viruses, Prokaryotes, and Eukaryotes’, *Annals of the New York Academy of Sciences*, 870: 100–7. 10415476 10.1111/j.1749-6632.1999.tb08870.x

[R17] Dudas G. , and Bedford T. (2019) ‘The Ability of Single Genes vs Full Genomes to Resolve Time and Space in Outbreak Analysis’, *BMC Evolution Biology*, 19: 232. 10.1186/s12862-019-1567-0 PMC6933756 31878875

[R18] Duffy S. , Shackelton L. A., and Holmes E. C. (2008) ‘Rates of Evolutionary Change in Viruses: Patterns and Determinants’, *Nature Reviews Genetics*, 9: 267–76. 10.1038/nrg2323 18319742

[R19] Gligic A. et al. (1988) ‘Hemorrhagic Fever with Renal Syndrome in Yugoslavia: Detection of Hantaviral Antigen and Antibody in Wild Rodents and Serological Diagnosis of Human Disease’, *Scandinavian Journal of Infectious Diseases*, 20: 261–6. 2900550 10.3109/00365548809032449

[R20] ——— (2008) ‘Etiology of Hemorrhagic Fever with Renal Syndrome, Viruses and Their Reservoirs’, In: Kovacevic, Z. et al. (eds) *Hemorrhagic Fever with Renal Syndrome*, pp. 17–34. Faculty of Medicine Kragujevac.

[R21] Hanada K. , Suzuki Y., and Gojobori T. (2004) ‘A Large Variation in the Rates of Synonymous Substitution for RNA Viruses and Its Relationship to a Diversity of Viral Infection and Transmission Modes’, *Molecular Biology and Evolution*, 21: 1074–80. 15014142 10.1093/molbev/msh109 PMC7107514

[R22] Heckel G. (2005) ‘Genetic Structure and Colonization Processes in European Populations of the Common Vole, *Microtus arvalis*’, *Evolution*, 59: 2231–42. 16405166

[R23] Heyman P. (2002) ‘Tula Hantavirus in Belgium’, *Epidemiology and Infection*, 128: 251–6. 12002543 10.1017/s0950268801006641 PMC2869818

[R24] Hofmann J. et al. (2021) ‘Tula Virus as Causative Agent of Hantavirus Disease in Immunocompetent Person, Germany’, *Emerging Infectious Disease*, 27: 1234–7. 10.3201/eid2704.203996 PMC8007307 33754997

[R25] Hughes A. L. , and Friedman R. (2000) ‘Evolutionary Diversification of Protein Coding Genes of Hantaviruses’, *Molecular Biology and Evolution*, 17: 1558–68. 11018161 10.1093/oxfordjournals.molbev.a026254

[R26] Jacob J. (2000) ‘Populationsökologische Untersuchungen an Kleinnagern Auf Unterschiedlich Bewirtschafteten Flächen der Unstrut‐Aue’, dissertation. Friedrich Schiller University Jena, Germany.

[R27] Jenkins G. M. (2002) ‘Rates of Molecular Evolution in RNA Viruses: A Quantitative Phylogenetic Analysis’, *Journal of Molecular Evolution*, 54: 156–65. 11821909 10.1007/s00239-001-0064-3

[R28] Jeske K. et al. (2021) ‘Hantavirus-*Leptospira* Coinfections in Small Mammals from Central Germany’, *Epidemiology and Infection*, 149: e97. 10.1017/S0950268821000443 PMC8101269 33612134

[R29] Jonsson C. B. et al. (2010) ‘A Global Perspective on Hantavirus Ecology, Epidemiology, and Disease’, *Clinical Microbiology Reviews*, 23: 412–41. 20375360 10.1128/CMR.00062-09 PMC2863364

[R30] Korva M. et al. (2013) ‘Phylogeographic Diversity of Pathogenic and Non-Pathogenic Hantaviruses in Slovenia’, *Viruses*, 5: 3071–87. 24335778 10.3390/v5123071 PMC3967161

[R31] Kumar S. et al. (2018) ‘MEGA X: Molecular Evolutionary Genetics Analysis across Computing Platforms’, *Molecular Biology and Evolution*, 35: 1547–9. 29722887 10.1093/molbev/msy096 PMC5967553

[R32] Kuzmina N. A. et al. (2013) ‘The Phylogeography and Spatiotemporal Spread of South-Central Skunk Rabies Virus’, *PLoS One*, 3: e82348. 10.1371/journal.pone.0082348 PMC3849458 24312657

[R33] Laenen L. et al. (2016) ‘Spatio-Temporal Analysis of Nova Virus, a Divergent Hantavirus Circulating in the European Mole in Belgium’, *Molecular Ecology*, 25: 5994–6008. 27862516 10.1111/mec.13887

[R34] ——— et al. (2019) ‘Identifying the Patterns and Drivers of *Puumala Hantavirus* Enzootic Dynamics Using Reservoir Sampling’, *Virus Evolution*, 5: vez009. 10.1093/ve/vez009 PMC6476162 31024739

[R35] Lemey P. et al. (2010) ‘Phylogeography Takes a Relaxed Random Walk in Continuous Space and Time’, *Molecular Biology and Evolution*, 27: 1877–85. 20203288 10.1093/molbev/msq067 PMC2915639

[R36] Ling J. et al. (2018) ‘Evolution and Postglacial Colonization of Seewis Hantavirus with *Sorex araneus* in Finland’, *Infection, Genetics and Evolution*, 57: 88–97. 10.1016/j.meegid.2017.11.010 29133028

[R37] Martin D. , and Rybicki E. (2000) ‘RDP: Detection of Recombination amongst Aligned Sequences’, *Bioinformatics*, 16: 562–3. 10980155 10.1093/bioinformatics/16.6.562

[R38] Nikolic V. et al. (2014) ‘Evidence of Recombination in Tula Virus Strains from Serbia’, *Infection, Genetics and Evolution*, 21: 472–8. 10.1016/j.meegid.2013.08.020 24008094

[R39] Papa A. (2012) ‘Dobrava-Belgrade Virus: Phylogeny, Epidemiology, Disease’, *Antiviral Research*, 95: 104–17. 22659378 10.1016/j.antiviral.2012.05.011

[R40] Parker J. , Rambaut A., and Pybus O. G. (2008) ‘Correlating Viral Phenotypes with Phylogeny: Accounting for Phylogenetic Uncertainty’, *Infection, Genetics and Evolution*, 8: 239–46. 10.1016/j.meegid.2007.08.001 17921073

[R41] Plyusnin A. et al. (1994) ‘Tula Virus: A Newly Detected Hantavirus Carried by European Common Voles’, *Journal of Virology*, 68: 7833–9. 7966573 10.1128/jvi.68.12.7833-7839.1994 PMC237245

[R42] ——— et al. (1995) ‘Genetic Variation in Tula Hantaviruses: Sequence Analysis of the S and M Segments of Strains from Central Europe’, *Virus Research*, 39: 237–50. 8837887 10.1016/0168-1702(95)00086-0

[R43] ——— (1996b) ‘Isolation and Characterization of Tula Virus, a Distinct Serotype in the Genus Hantavirus, Family Bunyaviridae’, *Journal of General Virology*, 77: 3063–7. 9000098 10.1099/0022-1317-77-12-3063

[R44] ——— et al. (1996a) ‘Hantaviruses: Genome Structure, Expression and Evolution’, *Journal of General Virology’*, 77: 2677–87. 8922460 10.1099/0022-1317-77-11-2677

[R45] Posada D. (2008) ‘JModelTest: Phylogenetic Model Averaging’, *Molecular Biology and Evolution*, 25: 1253–6. 18397919 10.1093/molbev/msn083

[R46] Pybus O. G. et al. (2012) ‘Unifying the Spatial Epidemiology and Molecular Evolution of Emerging Epidemics’, *Proceedings of the National Academy of Sciences of the United States of America*, 11: 15066–71. 10.1073/pnas.1206598109 PMC3443149 22927414

[R47] Rambaut A. et al. (2016) ‘Exploring the Temporal Structure of Heterochronous Sequences Using TempEst (Formerly Path-O-Gen)’, *Virus Evolution*, 9: vew007. 10.1093/ve/vew007 PMC4989882 27774300

[R48] ——— et al. (2018) ‘Posterior Summarisation in Bayesian Phylogenetics Using Tracer 1.7’, *Systematic Biology*, 67: 901–4. 29718447 10.1093/sysbio/syy032 PMC6101584

[R49] Ramsden C. et al. (2008) ‘High Rates of Molecular Evolution in Hantaviruses’, *Molecular Biology and Evolution*, 25: 1488–92. 18417484 10.1093/molbev/msn093

[R50] ——— et al. (2009) ‘Hantavirus Evolution in Relation to Its Rodent and Insectivore Hosts: No Evidence for Codivergence’, *Molecular Biology and Evolution*, 26: 143–53. 18922760 10.1093/molbev/msn234

[R51] Reynes J. M. et al. (2015) ‘Tula Hantavirus Infection in a Hospitalised Patient, France, June 2015’, *Euro Surveillance*, 20: 50. 10.2807/1560-7917.ES.2015.20.50.30095 26691901

[R52] Saxenhofer M. et al. (2017) ‘Revised Time Scales of RNA Virus Evolution Based on Spatial Information’, *Proceedings of the Royal Society B: Biological Sciences*, 284: 20170857. 10.1098/rspb.2017.0857 PMC5563803 28794221

[R53] ——— et al. (2019) ‘Secondary Contact between Diverged Host Lineages Entails Ecological Speciation in a European Hantavirus’, *PLoS Biology*, 17: e3000142. 10.1371/journal.pbio.3000142 PMC6382107 30785873

[R54] Schlegel M. et al. (2012) ‘Tula Virus Infections in the Eurasian Water Vole in Central Europe’, *Vector-Borne and Zoonotic Diseases*, 12: 503–13. 22225425 10.1089/vbz.2011.0784

[R55] Schmidt S. et al. (2021) ‘Spatial and Temporal Dynamics and Molecular Evolution of *Tula Orthohantavirus* in German Vole Populations’, *Viruses*, 13: 1132. 10.3390/v13061132 PMC8231151 34208398

[R56] Schmidt-Chanasit J. et al. (2010) ‘Extensive Host Sharing of Central European Tula Virus’, *Journal of Virology*, 84: 459–74. 19889769 10.1128/JVI.01226-09 PMC2798396

[R57] Schultze D. et al. (2002) ‘Tula Virus Infection Associated with Fever and Exanthema after a Wild Rodent Bite’, *European Journal of Clinical Microbiology and Infectious Disease*, 4: 304–6. 10.1007/s10096-002-0705-5 12072943

[R58] Schweizer M. et al. (2007) ‘Fine-Scale Genetic Structure and Dispersal in the Common Vole (*Microtus arvalis*)’, *Molecular Ecology*, 16: 2463–73. 17561906 10.1111/j.1365-294X.2007.03284.x

[R59] Sibold C. et al. (1995) ‘Genetic Characterization of a New Hantavirus Detected in *Microtus arvalis* from Slovakia’, *Virus Genes*, 10: 277–81. 8560789 10.1007/BF01701817

[R60] ——— et al. (1999) ‘Recombination in Tula Hantavirus Evolution: Analysis of Genetic Lineages from Slovakia’, *Journal of Virology*, 73: 667–75. 9847372 10.1128/jvi.73.1.667-675.1999 PMC103873

[R61] Sironen T. et al. (2001) ‘Molecular Evolution of Puumala Hantavirus’, *Journal of Virology*, 75: 11803–10. 11689661 10.1128/JVI.75.23.11803-11810.2001 PMC114766

[R62] Song J. W. et al. (2002) ‘Identification of Tula Hantavirus in Pitymys Subterraneus Captured in the Cacak Region of Serbia-Yugoslavia’, *International Journal of Infectious Diseases*, 6: 31–6. 12044299 10.1016/s1201-9712(02)90133-5

[R63] Souza W. M. et al. (2014) ‘Phylogeography and Evolutionary History of Rodent-Borne Hantaviruses’, *Infection Genetics and Evolution*, 21: 198–204. 10.1016/j.meegid.2013.11.015 24287104

[R64] Strimmer K. , and von Haeseler A. (1997) ‘Likelihood-Mapping: A Simple Method to Visualize Phylogenetic Content of a Sequence Alignment’, *Proceedings of the National Academy of Sciences of the United States of America*, 94: 6815–9. 9192648 10.1073/pnas.94.13.6815 PMC21241

[R65] Suchard M. A. et al. (2018) ‘Bayesian Phylogenetic and Phylodynamic Data Integration Using BEAST 1.10’, *Virus Evolution*, 4: vey016. 10.1093/ve/vey016 PMC6007674 29942656

[R67] Torres-Perez F. et al. (2011) ‘Spatial but Not Temporal Co-Divergence of a Virus and Its Mammalian Host’, *Molecular Ecology*, 20: 4109–22. 21880089 10.1111/j.1365-294X.2011.05241.x PMC3183239

[R68] Tougard C. et al. (2008) ‘New Insight into the Colonization Processes of Common Voles: Inferences from Molecular and Fossil Evidence’, *PLoS One*, 3: e3532. 10.1371/journal.pone.0003532 PMC2570793 18958287

[R69] Vapalahti O. et al. (1996) ‘Isolation and Characterization of Tula Virus, a Distinct Serotype in the Genus Hantavirus, Family Bunyaviridae’, *Journal of General Virology*, 77: 3063–7. 9000098 10.1099/0022-1317-77-12-3063

[R70] ——— et al. (2003) ‘Hantavirus Infections in Europe’, *The Lancet Infectious Diseases*, 3: 653–61. 14522264 10.1016/s1473-3099(03)00774-6

[R72] Wang M. (2012) ‘From Home Range Dynamics to Population Cycles: Validation and Realism of a Common Vole Population Model for Pesticide Risk Assessment’, *Health and Ecological Risk Assessment*, 9: 294–307. 10.1002/ieam.1377 23086922

[R0073a] Xia X. (2018) ‘DAMBE7: New and Improved Tools for Data Analysis in Molecular Biology and Evolution’, *Molecular Biology and Evolution*, 1: 1550–1552. 10.1093/molbev/msy073 PMC5967572 29669107

[R73] Yigit N. et al. (2016) ‘Microtus arvalis’, The IUCN Red List of Threatened Species 2016: e.T13488A22351133 <https://www.iucnredlist.org/>.

[R74] Zehender G. (2017) ‘Reconstructing the Recent West Nile Virus Lineage 2 Epidemic in Europe and Italy Using Discrete and Continuous Phylogeography’, *PLoS One*, 12: e0179679. 10.1371/journal.pone.0179679 PMC5497961 28678837

[R75] Zelena H. et al. (2013) ‘Tula Hantavirus Infection in Immunocompromised Host, Czech Republic’, *Emerging Infectious Diseases*, 19: 1873–5. 24209605 10.3201/eid1911.130421 PMC3837639

