# Supplementary material for: Phylogeographic analysis of Tula hantavirus highlights a single introduction to central Europe
Source: Virus Evol. 2022 Dec 22;8(2):veac112. doi: 10.1093/ve/veac112 (PMC10634634; doi:10.1093/ve/veac112)
Supplement: veac112_Supp [file veac112_supp.zip › suppl_data/Cirkovic_et_al_Supplementary data_R1.doc]

Figure S1

(a) The assessment of phylogenetic noise of the dataset through likelihood mapping method implemented in the program TreePuzzle, suggesting sufficient phylogenetic information within the alignment. (b) Estimation of substitution saturation level of the studied dataset test showing no saturation for closely related sequences compared to slight saturation for more distantly related sequences. The analysis was done in the software package DAMBE6.

Figure S2.

Time-scaled Bayesian maximum clade credibility (MCC) tree based on 412 TULV S segment sequences. In order to get better visibility of the tree, each branch was colored in accordance with the respective country. Clades consisting of two or more sequences originating from the same country are compressed. Error bars represent the 95% highest posterior density (HPD).
